# Cell Death, Molecular Targeted Therapies, and Metabolic Reprogramming in EGFR-Mutant Lung Cancer

**DOI:** 10.3390/cancers17172791

**Published:** 2025-08-27

**Authors:** Himani Joshi, M. Saeed Sheikh

**Affiliations:** Department of Pharmacology, State University of New York, Upstate Medical University, 750 E Adams Street, Syracuse, NY 13210, USA

**Keywords:** adenosine, Bim, epidermal growth factor receptor, metabolism, tyrosine kinase inhibitors, erlotinib, gefitinib, osimertinib

## Abstract

Lung cancer is responsible for high morbidity and mortality worldwide. In general, lung cancer can be divided into two major types, including small cell lung carcinoma (SCLC) and the more common non-small cell lung carcinoma (NSCLC). Molecular events underlying lung cancer development, growth, and progression remain complex. In addition to a variety of genetic aberrations, alterations in cellular metabolism have been implicated. Epidermal growth factor receptor (EGFR) is a cell surface protein that is frequently mutated in NSCLC. In this review, we discuss the effects of EGFR mutants on cell proliferative and survival signals, as well as metabolic reprogramming, in NSCLC. We also discuss the use and mechanisms of action of tyrosine kinase inhibitors (TKIs) that target EGFR-mutants and mediate their inhibitory effects by inducing cell death. Development of resistance to EGFR-TKIs is a problem in the clinic. We further discuss the approaches that are used to overcome this resistance, including the development of fourth-generation EGFR-TKIs. Immunotherapy is not very effective in EGFR-mutant NSCLC. We also discuss possible underlying mechanisms for the inadequate response of EGFR-mutant tumors to immunotherapeutics. Given that mutant EGFR transduces survival signals, and affects cellular metabolism, a better understanding of the crosstalk between mutant EGFR-mediated signals and metabolic reprogramming is expected to facilitate the development of newer personalized therapeutics to manage lung cancer.

## Simple Summary

Lung cancer remains a global health problem. Molecular pathogenesis of lung cancer is complex because many genetic and epigenetic abnormalities, as well as changes in cellular metabolism, are involved. Epidermal growth factor receptor (EGFR), a cell surface protein, is mutated in many lung cancers. In this review, we discuss the role of mutant EGFR in regulating lung cancer growth and cellular metabolism. We also discuss the use of small-molecule inhibitors that target the mutant EGFR for lung cancer treatment. Improved understanding of the underlying events by which mutant EGFR regulates cellular growth and metabolism is expected to facilitate the development of newer approaches to treat lung cancer.

## 1. Introduction

Lung cancer remains among the main contributors to cancer mortality, as most cases are diagnosed at advanced stages, which limits the effectiveness of treatment strategies [1]. Lung cancer can be divided into two major types: (i) small cell lung carcinoma (SCLC), which accounts for ~15% of all lung cancers, and (ii) non-SCLC (NSCLC), which is responsible for ~85% of all lung cancers [2]. NSCLC can be further subcategorized as adenocarcinoma (40%), squamous cell carcinoma (25–30%), and large cell carcinoma (10–15%) [2]. The development and progression of lung cancer are multi-step processes that involve continuous accumulation of genetic alterations over time. About 85% of NSCLC cases and ~98% of SCLC cases are linked to tobacco smoke [2]. Tobacco smoke contains numerous carcinogens, some of which are polycyclic aromatic hydrocarbons and nicotine-derived nitroso-aminoketones that form a complex with DNA, eventually leading to genetic aberrations [3,4]. Cytochrome P450 series of enzymes and glutathione-S-transferase enzymes are responsible for the metabolic activation of these carcinogens, which leads to the formation of a DNA complex. Consistent formation of DNA complexes with these carcinogens is believed to give rise to genetic changes [3,4]. A prominent feature of lung cancer is its diverse genetic mutations affecting key signaling pathways [3,5]. New technologies have led to greater improvements in identifying potentially targetable mutations in lung cancers. Molecular alterations in lung cancer have been identified in (i) growth factor receptors such as epidermal growth factor receptor (EGFR) and fibroblast growth factor receptor 1 (FGFR1), (ii) oncogenes including *K-RAS*, *B-RAF*, *DDR2*, and *PIK3CA,* and (iii) tumor suppressor genes such as *p53*, *LKB1*, and *PTEN*. Genetic rearrangements or translocations in important markers such as *ALK, ROS1*, and *RET* have also been reported [6]. Other markers, such as a monoglyceride lipase and PDRG1, are also deregulated in lung cancer [7,8,9]. In addition, deregulation of components of cell death pathways has also been linked to molecular pathogenesis of lung cancer [10].

Considerable variability exists in incidence of genetic mutations/alterations in various markers in NSCLCs. For example, *EGFR* mutations have been reported in about 32% cases of NSCLC, and *K-RAS* mutations in 25–30% of NSCLC cases [11]. Genetic mutations and alterations, albeit less frequent, have also been noted in *ALK* (3–7%), *BRAF* (2–4%), *MET* (3–5%), *RET* (1–2%), and *ROS1* (1–2%), [11]. Management of NSCLC involves surgery, chemotherapy, radiotherapy, targeted therapy, and immunotherapy (Table 1). Targeted therapies have been developed against various defective markers. In the case of mutant EGFR, tyrosine kinase inhibitors (TKIs) such as afatinib, dacomitinib, erlotinib, gefitinib, and osimertinib are approved. Alectinib, brigatinib, ceritinib, crizotinib, and lorlatinib are approved as ALK inhibitors. Dabrafenib with trametinib are approved for BRAF mutant NSCLCs. For MET-mutant tumors, capmatinib and tepotinib are approved. Pralsetinib and selpercatinib are approved for tumors harboring RET mutations. Crizotinib and entrectinib are for ROS1-positive tumors. Mutant K-Ras was undruggable for a long time; however, more recently, two K-Ras inhibitors have been approved: sotorasib and adagrasib [12]. The development of the first K-Ras inhibitors was a landmark advance. However, there remains a limitation in the use of K-Ras inhibitors because they inhibit only the G12C variant of K-Ras but not the other mutant variants [13].

## 2. Metabolic Reprogramming and Cancer

Lung cancer development is greatly influenced by genetic abnormalities. However, studies in the past several years have established that metabolic reprogramming also plays a significant role [14,15]. Rewiring of crucial metabolic pathways in cancer cells, including lung cancer, was recognized a century ago [16,17]. In the past two decades, extensive research has shown that cancer cells manipulate and reprogram their metabolic pathways, thus favoring tumor growth and metastasis [16,17,18]. Some distinct and notable characteristics of metabolic reprogramming in cancers include aerobic glycolysis, glutamine catabolism, use of citric acid cycle intermediates for energy production, and alterations in metabolite-driven gene regulation [19]. The first evidence of metabolic rearrangement in cancer cells was reported by Otto Warburg [17]. That landmark discovery opened the path to understanding how cancer cells utilized metabolic and energetic shifts for their growth, survival, and sustenance. Initial studies revealed that cancer cells upregulated glucose metabolism by increasing glucose uptake, upregulating glucose transporters and glycolytic enzymes [20]. Thus, for a long time, glucose metabolism remained at the center of metabolic rewiring in cancer cells. However, a large body of evidence now shows that glutamine catabolism is also crucial for cancer cells. Glutamine is one of the most abundant non-essential amino acids, and it is exploited by cancer cells in different ways [21]. Glutamine is the precursor of glutamate, which is then used to generate alpha-ketoglutarate and glutathione [21]. Alpha-ketoglutarate is used by cancer cells to replenish the TCA cycle intermediates through the process of anaplerosis, while glutathione primarily helps in reducing oxidative stress [21]. Therefore, glutamine plays a multifaceted role in metabolic reprogramming in cancer cells. In addition to certain nutrients, genes also play a role in influencing metabolic rewiring in cancer cells. Nuclear factor erythroid 2-related factor 2 (Nrf2), a key regulator of antioxidant response, also mediates metabolic reprogramming in lung cancer [22]. Some of the ways in which Nrf2 promotes metabolic rewiring are via increasing glutaminolysis, upregulation of genes that sustain PI3K/Akt signaling, and regulation of mitochondrial metabolism [22,23]. The multiple mechanisms through which Nrf2 controls metabolic reprogramming in cancer cells also make it an attractive target for the treatment and management of NSCLC.

Similar to other cancer types, metabolic reprogramming and dependence on glutamine have also been reported in NSCLC. One study reported that *K-RAS*-mutant NSCLC exhibits higher basal glutaminolysis than wild-type *K-RAS* NSCLC [24]. Consequently, targeted inhibition of glutaminolysis using CB-839 enhanced antitumor activity of selumetinib (MAPK/ERK inhibitor) in *K-RAS*-mutant NSCLC [24]. Along these lines, another study reported that glutaminase inhibition by CB-839 sensitized human lung cancer xenografts to radiation therapy in mice [25]. CB-839 is a small molecule inhibitor that inhibits glutaminase-I, the enzyme responsible for conversion of glutamine to glutamate [26]. Supinoxin is another small molecule that inhibits the p68 RNA helicase (DDX5) [27]. Das et al. [27] reported that supinoxin results in mitochondrial dysfunction in chemo-resistant SCLC cells, thereby inhibiting their growth in vitro and in vivo [27]. In addition to small molecules that target cancer cell metabolism, there are also certain tumor markers or proteins that play a role in metabolic rewiring and, thus, can be used as prognostic markers. For instance, SLC7A11 protein overexpression in NSCLC was found to be correlated with increased glutamine dependence, as well as worse 5-year survival [28]. Therefore, such markers that regulate the metabolic shifts in cancer cells have the potential to be developed into therapeutic targets.

## 3. Epidermal Growth Factor Receptor (EGFR)-Mediated Signaling and Metabolic Reprogramming

**Figure 1:**
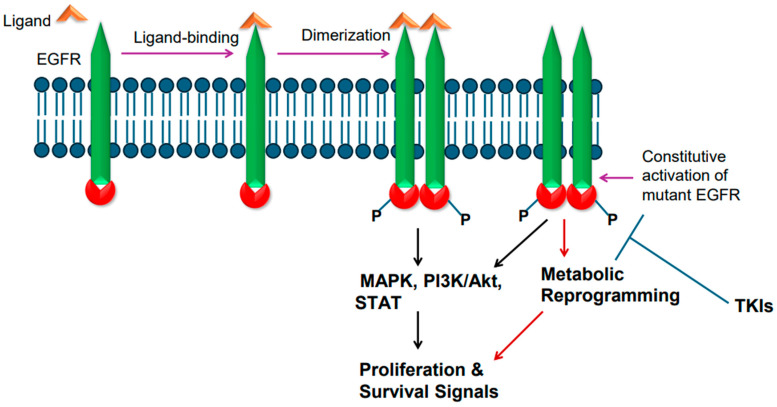
Epidermal growth factor receptor (EGFR) activation and signaling. Wild-type EGFR is activated by its ligand, whereas mutant EGFR is constitutively active. Ligand binding in the extracellular region induces receptor dimerization and tyrosine phosphorylation of intracellular tyrosine kinase domain. The mutant EGFR is constitutively active. The active receptor transduces proliferative and survival signals.

Epidermal growth factor receptor (EGFR), a single-chain transmembrane receptor, belongs to the erbB family [14]. In addition to EGFR (also known as erbB1), three additional receptors exist in this family: erbB2 (HER2), erbB3, and erbB4 [29]. Except for erb3, all these receptors harbor tyrosine kinase activity due to the presence of an intracellular tyrosine kinase domain. EGFR is activated by various ligands that bind to its extracellular region. Some of the EGFR ligands include epidermal growth factor (EGF), transforming growth factor alpha (TGFa), amphiregulin (AREG), heparin-binding EGF-like growth factor (HBEGF), epigen (EPGN), and epiregulin (EREG) [30]. Ligand binding to EGFR leads to ligand-induced dimerization, which results in phosphorylation-mediated tyrosine kinase activation, culminating in transduction of cell proliferation and survival signals via RAS/RAF/ERK-dependent signaling pathway [31]. EGFR-mediated signaling also leads to activation of PI3K/AKT/mTOR and STAT-mediated signaling events [31] (Figure 1).

*EGFR* is frequently mutated in NSCLC. The incidence of *EGFR* gene mutations is higher in Asian populations compared to people in Western countries [3,32,33]. Approximately 62% of adenocarcinomas also exhibit EGFR protein overexpression [3,32,33]. In general, mutations in *EGFR* gene occur in exons 18–21, a region that codes for the receptor tyrosine kinase domain [34]. Small deletions in exon 19 are the most common; point mutation in exon 21 changing leucine at position 858 to arginine [L858R] is also common [34]. Exon 20 insertion mutations have also been found [35]. The T790M mutation changing threonine to methionine at amino acid position 790 in the tyrosine kinase domain is also reported [36]. The T790M mutation is considered a secondary type of mutation as it is acquired after treatment with tyrosine kinase inhibitors (TKIs). The mutant EGFR is believed to become constitutively active and transduce proliferative and survival signals, conferring growth advantage upon cancer cells (Figure 1). Upon constitutive activation, the mutant *EGFR* activates the canonical signaling pathways, such as RAS/RAF/ERK-dependent and PI3K-mediated signaling events (Figure 1). In addition, the constitutively active mutant *EGFR* also plays a role in metabolic reprogramming (Figure 1), particularly in lung adenocarcinomas, as enhanced glucose uptake and more lactate formation have been reported [37,38]. Furthermore, increased regulation of pentose phosphate pathway and pyrimidine biosynthesis are reported as well [37,38]. *EGFR* mutations in NSCLC were found to increase glycolysis, which was needed to stabilize EGFR [39].

## 4. EGFR as a Target of Therapeutics

**Figure 2:**
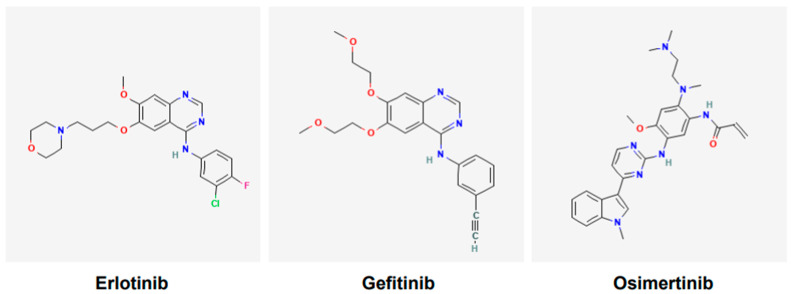
Chemical structures of tyrosine kinase inhibitors. Structures of gefitinib, erlotinib, and osimertinib are according to PubChem. Gefitinib and erlotinib are first-generation EGFR tyrosine kinase inhibitors (TKIs), whereas osimertinib is the third-generation EGFR TKI.

Various tyrosine kinase inhibitors (TKIs) targeting EGFR have been developed and include gefitinib, erlotinib, icotinib, afatinib, dacomitinib, and osimertinib. Gefitinib (Figure 2) was the first TKI of EGFR to receive the United States Food and Drug Administration (USFDA)’s approval for the treatment of lung cancer in 2015 [40]. However, it was reported following various clinical trials that only a subgroup of patients of Asian ethnicity benefited from the gefitinib and chemotherapy combination; the other cohorts did not [41]. Currently, osimertinib (Figure 2), a third-generation TKI, is a first-line option for NSCLC harboring EGFR mutations [42]. It is also effective against tumors that have acquired T790M mutations [42]. Osimertinib is used orally, it is approved only for adults, and has no contraindications linked to its use.

According to the United States Food and Drug Administration (USFDA)-approved drug label (updated as of September 2024), osimertinib (brand name Tagrisso) is indicated for EGFR-mutant NSCLCs, specifically for tumors harboring exon 19 deletions or exon 21 L858R mutations. Its use is indicated as follows: (i) in adjuvant setting following resection of tumor. (ii) For locally advanced, unresectable tumors (stage III) that have progressed during or after chemoradiation, including platinum-based regimens. (iii) As a first-line option for metastatic tumors. (iv) As a first-line option in combination with pemetrexed and platinum-based regimen for locally advanced or metastatic disease. (v) For metastatic tumors with EGFR T790M mutation that have progressed on other EGFR-TKIs. The warnings and precautions according to the label include interstitial lung disease (ILD)/pneumonitis, QTc interval prolongation, cardiomyopathy, erythema multiforme major, Stevens–Johnson syndrome, toxic epidermal necrolysis, cutaneous vasculitis, aplastic anemia, and embryo–fetal toxicity. The common side effects according to the label are as follows: (i) as a single agent—leukopenia, lymphopenia, thrombocytopenia, anemia, diarrhea, rash, musculoskeletal pain, neutropenia, nail toxicity, dry skin, stomatitis, and fatigue. (ii) If given as a single agent after platinum-based chemoradiation therapy—lymphopenia, leukopenia, ILD/pneumonitis, thrombocytopenia, neutropenia, rash, diarrhea, nail toxicity, musculoskeletal pain, cough, and COVID-19. (iii) If used in combination with pemetrexed and platinum-based regimen—leukopenia, thrombocytopenia, neutropenia, lymphopenia, rash, diarrhea, stomatitis, nail toxicity, dry skin, and increased blood creatinine.

**Figure 3:**
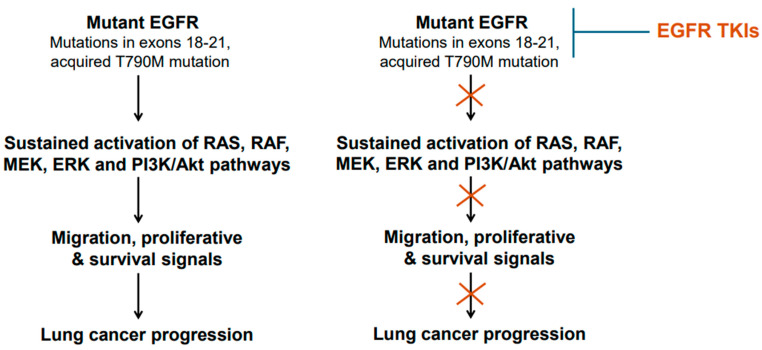
Mutant epidermal growth factor receptor (EGFR) activation and inhibition. Common mutations/genetic alterations reside in exons 18–21 of the EGFR gene. This region corresponds to tyrosine kinase domain. T790M mutation is a secondary mutation as it is acquired during the course of treatment with early-generation EGFR TKIs. NSCLCs harboring T790M mutation are responsive to osimertinib.

The mechanism of action of the EGFR-TKIs involves binding to the ATP-binding site in the tyrosine kinase domain of the mutant EGFR [43]. After binding the mutant EGFR, the TKIs interfere with EGFR phosphorylation and consequently block the transduction of proliferative and survival signals (Figure 1 and Figure 3). Thus, the TKIs are ATP-competitive inhibitors. Of these TKIs, the first-generation ones, such as gefitinib, erlotinib, and icotinib, reversibly bind at the ATP-binding site [43]. The second-generation TKIs, including afatinib and dacomitinib, irreversibly bind at the ATP-binding site [43]. The third-generation TKI osimertinib also binds irreversibly at the ATP-binding site [43]. Notably, the cysteine residue at amino acid position 797 within the ATP-binding region of the EGFR is important for osimertinib binding [43]. Because tumors harboring T790M mutant-EGFR developed resistance against earlier generations of TKIs, osimertinib was developed to counter the T790M-linked resistance, and at the same time target the EGFR-sensitizing mutations [42,43]. An additional feature of osimertinib is its ability to cross the blood–brain barrier and, thus, it is suitable for tumors with brain metastases. Overall, osimertinib has proven to be more selective and potent, and exhibits less toxicity compared to early generation TKIs that were developed against mutant EGFR [43]. Accordingly, it is a first-line option for NSCLCs harboring EGFR mutations.

Cellular metabolism also plays a role in regulating the sensitivity and resistance of lung cancer cells to the EGFR-TKIs. The EGFR-TKIs have also been reported to interfere with mutant EGFR-mediated metabolic reprogramming. It was noted that treatment with TKIs gefitinib and erlotinib led to a decrease in lactate production and glucose consumption in lung adenocarcinoma cells. Metabolomic analysis further revealed that after treatment with these TKIs, metabolites of glycolytic and pentose phosphate pathway (PPP) decreased significantly in lung adenocarcinoma cells [38]. Osimertinib-induced growth inhibition and glycolysis inhibition have also been reported in osimertinib-sensitive cell lines [44,45]. It was noted that in NSCLC cells, EGFR mutations increased glycolysis, which was needed to stabilize EGFR [39].

Lung cancer cells are believed to also acquire resistance to EGFR-TKIs via metabolic reprogramming. In this context, metabolic profiles of erlotinib-sensitive and -resistant cells were analyzed, and the results indicated differences in various metabolites, including glycolytic intermediates between the sensitive and resistant cell lines [45,46]. Metabolomics-mediated analysis of osimertinib-sensitive and -resistant cells also revealed different rates of abundance of various metabolites in sensitive and resistant cells [47,48]. In a preclinical study, it was reported that although osimertinib inhibited glycolysis in lung cancer with EGFR mutations, it did not do so in osimertinib-resistant cells [44]. It was further reported that osimertinib appeared to rely on mitochondrial oxidative phosphorylation (OXPHOS), and OXPHOS inhibitors delayed or prevented the development of resistance to osimertinib [44].

## 5. Cell Death Induced by EGFR-TKIs

EGFR-TKIs induce cell death to mediate their anticancer effects. The mechanisms of TKI-induced cell death involve engagement of both the intrinsic and extrinsic pathways of cell death. In preclinical studies, gefitinib was reported to induce cell death in lung cancer cell lines harboring mutant *EGFR* but not wild-type *EGFR* [48]. Gefitinib-induced apoptosis engaged intrinsic pathway of apoptosis and involved Bax activation, as well as classical features such as activation of the caspase cascade and PARP cleavage. Furthermore, gefitinib induced upregulation of Bim in a time- and dose-dependent manner. Bim is a BH3-only proapoptotic member of Bcl-2 family. Interestingly, gefitinib did not affect other BH3-only members of the Bcl-2 family, such as PUMA, BAD, and BMF, nor the pro-survival members, including BCL-w, BCL-xL, and MCL1 [48]. Further investigation revealed that Bim was needed for gefitinib-induced apoptosis in the lung cancer cell lines in the study [45]. Costa et al. [49] also reported that Bim was essential in gefitinib-mediated cell death in EGFR-mutant lung cancer cells.

**Figure 4:**
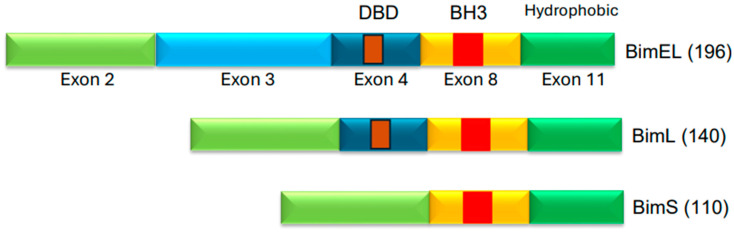
Three isoforms of Bim protein. BimEL (extra-long), BimL (long), and BimS (short) are the common Bim isoforms [49,50]. The protein regions corresponding to exons are indicated. BH3 domain is present in the protein region encoded by exon 8. DBD (Dynein Binding Domain) is in the region encoded by exon 4. The DBD is believed to facilitate interactions with dynein light chain 1 (DLC1) [54].

Another study, by Gong et al. [50], used erlotinib and reported that erlotinib also engaged intrinsic pathway, and that Bim was required for erlotinib-mediated cell death in lung cancer cell lines harboring *EGFR* mutations. Deng et al. [51] also independently found erlotinib-induced apoptosis to be linked to Bim upregulation. Thus, several independent studies found Bim to be an important mediator of apoptosis induced by EGFR-TKI. A number of isoforms of Bim exist, but the predominant isoforms are BimEL (extra-long), BimL (long), and BimS (short) (Figure 4). These isoforms are generated via alternative splicing [52,53]. BimEL is composed of 196 amino acids, BimL has 140 amino acids, and BimS is made of 110 amino acids (Figure 4). The BH3 domain is present in all isoforms; however, only BimEL and BimL harbor the dynein binding domain (DBD). All Bim isoforms are believed to function as proapoptotic proteins. Bim is believed to mediate its apoptotic effects by interacting with the anti-apoptotic members of the Bcl-2 family via its BH3 domain and thereby neutralize their anti-apoptotic effects. Bim is also noted to mediate its pro-apoptotic effects by interacting with Bax in order to affect Bax conformation and, consequently, Bax activation. ABT-737 is a BH3-mimetic small molecule that is known to inhibit Bcl-2 and Bcl-XL. ABT-737 was also used in EGFR-mutant lung cancer cells and was found to potentiate the apoptotic effect of gefitinib and erlotinib [48,50].

The third-generation osimertinib (AZD9291) has also been shown to induce apoptosis in lung cancer cells that harbor EGFR mutations. Osimertinib-induced apoptosis involved activation of caspases 8 and 3 and PARP cleavage [55]. Furthermore, osimertinib-induced cell death was also coupled with Bim upregulation and downregulation of Mcl-1. Although osimertinib did not alter the Bax and Bcl-2 levels, it nevertheless inhibited the phosphorylation of ERK, Bim, and Mcl-1. Osimertinib did not induce apoptosis or mediate similar effects in osimertinib-resistant cells. Mechanistic investigation revealed that osimertinib enhanced Bim levels by inhibiting its degradation, whereas it promoted Mcl-1 degradation via proteasomal pathway. It was also reported that Bim upregulation and Mcl-1 suppression were needed for osimertinib to mediate its apoptotic effects in EGFR-mutant lung cancer cells [55].

Development of osimertinib resistance also occurs. Using osimertinib-resistant lung cancer cells, strategies to overcome resistance have been reported in preclinical studies. Shi et al. [55] studied the effect of MEK inhibitors in combination with osimertinib in osimertinib-resistant lung cancer cells. They noted that MEK inhibitors alone or osimertinib alone did not induce considerable cell death in osimertinib-resistant cells. However, when both MEK inhibitor and osimertinib were combined, significant cell death was induced, which was noted both in vitro and in vivo [55]. Ma et al. [56] reported that targeting intrinsic pathway of cell death can be a strategy to overcome osimertinib resistance. They reported that triple combination with Mcl-1 inhibitor, Bax activator, and osimertinib was the most effective in inducing cell death in osimertinib-resistant cells and, thus, also mediating anticancer effect in an in vivo setting.

Osimertinib and other TKIs have also been reported to induce cell death by engaging the extrinsic pathway of apoptosis [57]. Shi et al. [58] reported that osimertinib, erlotinib, and afatinib inhibited the levels of cFLIP. cFLIP is a truncated version of caspase 8/10 homologue that inhibits extrinsic pathway of cell death. By suppressing cFLIP, these TKIs were believed to activate the extrinsic pathway of cell death in EGFR-mutant lung cancer cells [58].

## 6. Osimertinib Resistance and Fourth-Generation EGFR-TKIs

As noted above, osimertinib, a third-generation TKI, is a first-line option for NSCLC with mutant *EGFR* [39]. It is an aminopyrimidine-based irreversible EGFR-TKI [59]. It is also used for tumors that have acquired T790M mutations [39]. Central nervous system (CNS) metastases are common and indicate poor prognosis in NSCLC patients carrying EGFR mutations [60]. Osimertinib can cross the blood–brain barrier; accordingly, it has shown effectiveness in CNS metastases [60,61]. Despite its promise as an effective third-generation EGFR-TKI, acquired resistance to osimertinib has been reported [61]. The mechanism underlying osimertinib resistance is multifaceted in nature and reported to be either EGFR-dependent or -independent [61]. The EGFR-independent mechanism of osimertinib resistance is complex and involves various signaling pathways. Some approaches to overcome osimertinib resistance are mentioned in Section 4 of this article.

In the case of EGFR-dependent resistance mechanisms, mutations in the C797 residue of the EGFR protein play a critical role [59]. In context of osimertinib’s mechanism of action, the cysteine at position 797 (C797) is important in facilitating its binding within the ATP-binding region of the kinase domain of EGFR. The C797 residue is encoded by the nucleotides residing in exon 20 of the *EGFR* gene. Osimertinib interacts covalently with the mutant EGFR via the cysteine residue at position 797 [59]. There is a degree of selectivity in that osimertinib predominantly interacts with the mutant EGFR, and thus only minimally affects the wild-type version. This selectivity is believed to underlie its effectiveness and lower toxicity than the earlier-generation EGFR-TKIs [59].

Mutations in C797 residue have been noted in several osimertinib-resistant cases. C797S mutation is more common and has been reported in the first- or second-line use of osimertinib [61]. Because cysteine is replaced by serine at position 797, the interactions between the mutant EGFR and osimertinib are lost. In some cases of acquired resistance to osimertinib, the T790M mutation is retained, whereas in some other cases, the T790M mutation is lost [61]. The context of C797S co-occurring mutation with T790M, either in cis or trans, is important in relation to osimertinib resistance [61,62,63,64]. If the C797S is in cis then the cells harboring both C797S and T790M are resistant to all types of EGFR-TKIs. However, if C797S is in trans relative to T790M, then cells remain sensitive to quinazoline-based EGFR-TKIs such as gefitinib and erlotinib [61,62,63,64,65]. The C797 residue is critical for osimertinib function; accordingly, C797S is a common mutation in relation to development of osimertinib resistance [66]. Therefore, efforts have been expended to develop the fourth-generation EGFR-TKIs to overcome C797S-based osimertinib resistance. Several fourth-generation EGFR-TKIs have been tested in preclinical studies, and some of those are undergoing clinical testing.

**Figure 5:**
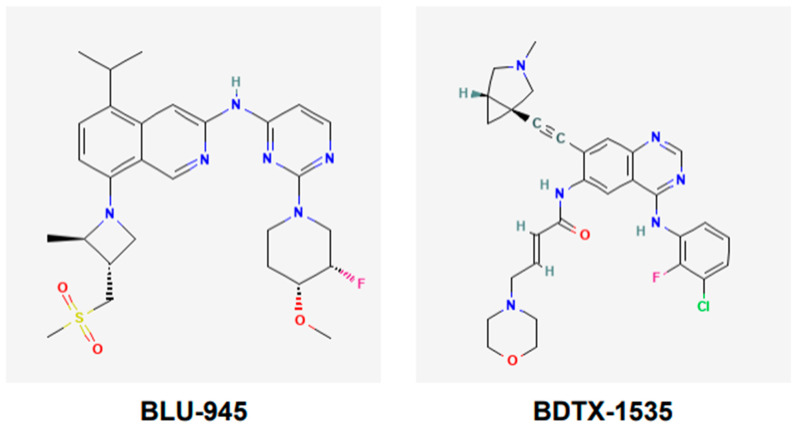
Chemical structures of fourth-generation tyrosine kinase inhibitors. Structures of BLU-945 and BDTX-1535 are according to PubChem. BLU-945 is an oral aminopyrimidine-based reversible fourth-generation orthosteric EGFR-TKI. It is a reversible inhibitor. BDTX-1535 is an oral quinazoline-based EGFR-TKI. It is an irreversible inhibitor that can penetrate the brain.

BLU-945, also known as tigozertinib, is an aminopyrimidine-based reversible fourth-generation orthosteric EGFR-TKI (Figure 5). In preclinical studies, it has shown promise as a single agent against osimertinib-resistant patient-derived xenografts [67,68,69]. It is effective against common EGFR-activating mutations, as well as C797S and T790M mutant versions of EGFR. BLU-945 is an oral agent that has been tested singly, and also in combination with osimertinib in a phase I/II SYMPHONY trial [67,68,69]. It was reported that 108 patients were given BLU-945 as monotherapy, whereas 25 patients were on a BLU-945 and osimertinib combination. The results indicated that BLU-945 singly and with osimertinib was tolerated well and demonstrated a decrease in circulating tumor DNA (ctDNA); tumor shrinkage was also noted [69]. It was further reported that BLU-945 in combination with osimertinib exerted responses at doses that were lower than those used for monotherapy [69]. As of January 2024, the drug sponsor (Blueprint Medicines, Cambridge, MA, USA) has decided to drop further clinical development of BLU-945 for this malignancy. However, BLU-945 remains a viable drug that can be further investigated for its potential in NSCLC.

BDTX-1535, also known as silevertinib, is an oral quinazoline-based EGFR-TKI (Figure 5). It is an irreversible inhibitor that is also a brain penetrant. BDTX-1535 is effective against common EGFR-activating mutations and acquired mutations, including C797S. Initial findings of phase II trial testing BDTX-1535 in NSCLCs carrying EGFR-activating mutations, including the C797S resistance mutation, were reported in August 2024. The overall response rate (ORR) was the primary endpoint of the trial. A 36% ORR was noted among the patients who met the protocol criteria. Of those patients whose tumors harbored osimertinib-resistance mutations, the ORR was reported to be 42%. Some of the secondary endpoints were to determine treatment-related adverse events (AEs), as well as the optimal dose. It was noted that those in the 200 mg dose cohort experienced mild to moderate AEs. The phase II trial is continuing and will further evaluate the potential of BDTX-1535 [70].

JIN-A02 is an orally available EGFR-TKI that is specific for common EGFR-activating mutations, as well as acquired mutations, including C797S mutation. It is believed to be specific for these mutations in both cis and trans orientations. JIN-A02 is a brain penetrant and has shown promise in preclinical studies; it is currently under phase I/II clinical investigation [71].

BPI-361175 is an EGFR-TKI that has shown specificity towards mutant EGFR, including C797S mutation. BPI-361175 is reported to spare the wild-type EGFR. Its promise has been demonstrated in preclinical studies involving in vitro as well as in vivo approaches. It is an oral agent that is also a brain penetrant. Currently, BPI-361175 is in phase I/II clinical trials [72].

TRX-221 is an orally available EGFR-TKI that is also specific for C797S mutant EGFR. It is capable of crossing the blood–brain barrier and has shown promise in preclinical studies against osimertinib-resistant xenografts carrying EGFR C797S mutation. TRX-221 is currently in phase I/II clinical trials [73].

BI-732 is an EGFR-TKI that is orally available. In preclinical studies involving in vitro and in vivo approaches, BI-732 has been reported to exhibit effectiveness against common EGFR-activating mutations, as well as acquired resistance mutations, including the C797S mutation. It has also been tested in combination with osimertinib and was reported to be effective at doses lower than those used for monotherapy. BI-732 is reported to be EGFR-mutant-selective, and it is also capable of crossing the blood–brain barrier [74].

TQB3002 is a more recent addition to the list of fourth-generation EGFR-TKIs. It is reported to inhibit various versions of EGFR-mutants, including the C797S mutant. It is reported to have shown superior activity compared to osimertinib in preclinical in vitro and in vivo studies. It is currently undergoing phase I trial [75].

## 7. Immunotherapy and Mutant EGFR

Immunotherapeutics such as immune checkpoint inhibitors, including monoclonal antibodies against programmed cell death protein 1 (PD-1) and its ligand PD-L1, have been approved in recent years to treat NSCLC. Various anti-PD-1 antibodies are currently in use, including nivolumab (Opdivo), pembrolizumab (Keytruda), and cemiplimab (Libtayo). The anti-PD-1L antibodies that are approved include atezolizumab (Tecentriq) and durvalumab (Imfinzi). The effectiveness of these checkpoint inhibitors in NSCLC remains variable and depends on other immune factors, such as the presence of CD8+ T cells and PD-1/PD-L1 expression in the tumor microenvironment (TME) [76]. In the case of EGFR-mutant NSCLC, these checkpoint inhibitors have not proven very effective [77,78].

For example, the KEYNOTE-010 phase II/III trial enrolled advanced NSCLC patients who did not benefit from platinum-based chemotherapy [79]. Furthermore, the tumors of enrolled patients were expected to have PD-L1 tumor proportion scores (TPSs) of 1% or higher and no prior treatment with docetaxel. Those with NSCLC harboring mutant EGFR were expected to have failed TKI therapy. The patients were randomized and given pembrolizumab 2 mg/kg, pembrolizumab 10 mg/kg, or docetaxel 75 mg/m^2^. The primary endpoints included overall survival (OS) and progression-free survival (PFS). The results indicated that in general, patients in this population responded favorably to pembrolizumab. However, the subgroup of patients with tumors harboring EGFR mutations did not benefit from pembrolizumab [79]. In the CheckMate 057 phase III trial that evaluated nivolumab compared to docetaxel in non-squamous NSCLC patients previously treated with platinum-based chemotherapy, the subgroup of patients with EGFR mutations also did not show benefit from nivolumab [80]. The POPLAR phase 2 trial evaluated NSCLC patients who had also failed previous platinum-based chemotherapy [81]. Patients were randomized and treated with atezolizumab (anti-PD-L1) or docetaxel. Overall survival (OS) was the primary endpoint of the trial. The results indicated that atezolizumab was tolerated well and provided survival benefit when compared to docetaxel. However, the subgroup of patients with EGFR mutations did not experience significant benefits from atezolizumab when compared to docetaxel [81]. The Phase III Keynote-789 study investigated the effect of combining pembrolizumab with carboplatin in stage IV non-squamous NSCLC patients who had previously been treated with TKIs. The results indicated that in NSCLC patients carrying mutant EGFR with acquired TKI-resistance, pembrolizumab addition did not significantly prolong overall or progression-free survival [82].

Currently, National Comprehensive Cancer Network (NCCN) clinical practice guidelines do not recommend immunotherapeutics for treating EGFR-mutant NSCLC [77]. This is primarily due to the diminished effectiveness of PD-1/PD-L1 inhibitors in EGFR-mutant NSCLC. It is not entirely clear why the checkpoint inhibitors are not very effective in EGFR-mutant NSCLC. The EGFR-mutant NSCLCs are considered “immunologically cold” because these tumors are believed to have immunosuppressive microenvironments. Various possibilities have been proposed regarding the underlying mechanism(s) for the poor response of EGFR-mutant tumors to immunotherapeutics. Although this remains to be fully investigated, the lung cancers with mutant EGFR tend to have fewer tumor-infiltrating lymphocytes (TILs), such as CD8+ T cells [78]. Furthermore, the antigen-presenting dendritic cells (DCs) that are important for T cell activation were reported to have their function compromised in the EGFR-mutant TME. Tregs are immunosuppressive cells that are suggested to be deregulated in EGFR-mutant TME [78].

Alterations in cellular metabolism in EGFR-mutant tumors are also believed to play a role in creating immunosuppressive TME. In this context, the metabolic conversion of ATP into adenosine and adenosine’s role in creating immunosuppressive TME is notable [83,84]. For example, excessive ATP, when generated due to chronic inflammation, hypoxia, and cellular turnover in TME, sets the stage for activation of metabolic pathway involving ectonucleotidases CD39 and CD73, which act upon ATP. The ectonucleotidase CD39 generates ADP and AMP after hydrolyzing ATP. The ectonucleotidase CD73 eventually converts AMP into adenosine [83,84].

**Figure 6:**
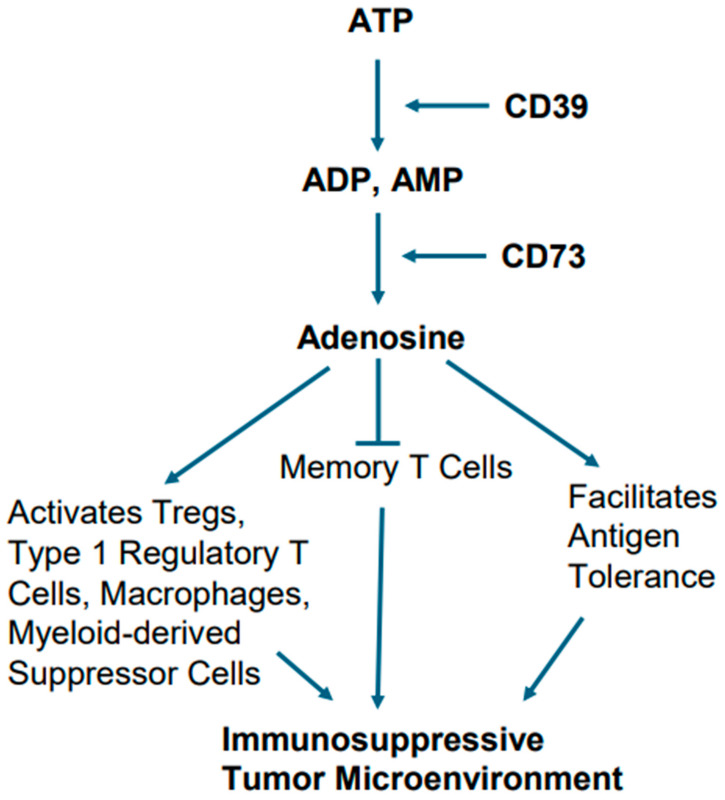
Metabolic pathway of adenosine generation and consequent immunosuppression. Hydrolysis of ATP by the ectonucleotidases CD39 and CD73 ultimately leads adenosine generation. Adenosine potentiates the immune-suppressive actions of Tregs, type 1 regulatory T cells, macrophages, and myeloid-derived suppressor cells, and also facilitates antigen tolerance, as well as blocking memory T cells from becoming effector cells.

Adenosine has been implicated in a variety of cellular processes by activating both the high-affinity and low-affinity receptors. Adenosine as an immune modulator potentiates the immune-suppressive actions of Tregs, type 1 regulatory T cells (Tr1), macrophages, and myeloid-derived suppressor cells (MDSCs). Furthermore, it facilitates antigen tolerance and blocks memory T cells from becoming effector cells (Figure 6). Collectively, these effects create a TME that promotes tumor growth and is not conducive to immunotherapy [83,84].

Studies are currently ongoing to restrain adenosine’s immunosuppressive effects to enhance the effect of immunotherapy in mutant EGFR lung cancers. Various approaches are being used. Notable among these are the use of monoclonal antibodies against CD39 and CD73 to inhibit adenosine generation [78,83,84]. Antagonists of adenosine receptors are also being tested. It is notable that anti-CD73 monoclonal antibody, namely olecumab, is being evaluated in combination with checkpoint inhibitors [78,83,84]. The COAST study, a phase II trial, has investigated the durvalumab (anti-PD-L1) and olecumab (anti-CD73) combination in unresectable stage III NSCLC [85]. Although the EGFR status of the tumors in this trial is not known, the outcome indicated that durvalumab plus olecumab worked favorably to enhance overall response rate (ORR) and progression-free survival (PFS) when compared to durvalumab monotherapy [85]. The PACIFIC-9 study, a phase III trial exploring durvalumab plus oleclumab or monalizumab in unresectable stage III NSCLC, is currently ongoing [86].

Macroautophagy, commonly referred to as autophagy, is the process that involves degradation of damaged organelles, misfolded proteins, or micro-organisms through lysosomal actions [87]. Therefore, once completed, autophagy leads to generation of nucleotides, amino acids, fatty acids, sugars, and ATP [87]. Autophagy is activated by various stimuli such as nutrient starvation, oxidative stress, hypoxia, DNA damage, and pathogens [87,88]. Conventionally, autophagy is a process that ensures quality control and maintains homeostasis [87]. However, in the case of cancers, autophagy can either suppress tumor growth or contribute to the progression of cancer [87]. In the last two decades, a large body of evidence has shown that autophagy can promote cancer progression as it generates nutrients and amino acids, thereby relieving nutrient stress in cancer cells, and also reducing inflammation [88]. Interestingly, some studies have reported that autophagy could lead to an immunosuppressive tumor microenvironment [89]. For instance, Carleton et al. [90] reported that CD8+ T cells lacking Atg5 produce more interferon gamma and tumor necrosis factor. Another study, by Wang et al. [91], demonstrated that inhibition of autophagy in gastric cancer (in vivo and in vitro) led to upregulation in interferon gamma and PD-L1 via the p62/SQSTM1-NF-κB pathway. It is possible that activation of autophagy could also contribute to an immunosuppressive microenvironment in EGFR-mutant NSCLCs. Therefore, inhibiting autophagy may increase the susceptibility of certain cancers, including EGFR-mutant cancers, to immunotherapeutics.

## 8. Conclusions

Lung cancer is a global health problem. Advances made in biomedical research are being translated into the clinic with the goal of improving the management of lung cancer. Molecular pathogenesis of lung cancer is complex; in addition to genetic changes, alterations in cellular metabolism also play a role. EGFR mutations are common in NSCLCs. Mutant EGFR becomes constitutively active and plays an important role in lung cancer pathogenesis. Clearly, mutant EGFR is an important target of cancer therapeutics. The EGFR-TKIs have now become part of the standard of care for EGFR-mutant NSCLCs. Although the development and use of EGFR-TKIs have improved the management of lung cancer in the clinic, toxicity of these agents and development of resistance are of concern. Furthermore, EGFR-mutant NSCLCs have immunosuppressive TME; accordingly, immunotherapy is not very effective in subgroups of NSCLC patients that harbor EGFR mutations. In this article, we have discussed the molecular events that are linked to tumor progression and rewiring of metabolic pathways in EGFR-mutant NSCLCs. We have also discussed deregulation of cell death and its relationship to acquisition of resistance to EGFR TKIs currently used in the clinic. Outcomes of clinical trials focused on investigating the effectiveness of TKIs and the ineffectiveness of immunotherapy in EGFR-mutant NSCLCs are also discussed. By analyzing the current state of knowledge on these topics, this article highlights the scientific gaps that ongoing and future research will bridge to improve the management of NSCLC. Because cellular metabolism also influences cancer growth and progression, future development of therapeutics is expected to take an integrated approach focusing on genetic and immune markers, as well as cellular metabolism.

## Figures and Tables

**Table 1:** Current strategies to manage non-small cell lung cancer according to American Cancer Society.

Stage	Description	Treatment Strategy
I	No primary tumor, or tumor 3–4 cm with no regional or distant metastases	Surgery (segmental resections) Radiation in patients who cannot have surgery (stereotactic body radiotherapy for patients in whom cancer is confined to lungs) Chemotherapy after surgery in patients with tumor ≥ 4 cm (cisplatin and vinorelbine)
II	Tumor 4–7 cm with either no regional node metastases or ipsilateral pulmonary node metastases, but no distant metastases	Surgery (segmental resections) Radiation in patients who cannot have surgery Chemotherapy in patients whose cancer has spread to bronchial lymph nodes (cisplatin and vinorelbine)
III	Metastases in ipsilateral pulmonary nodes, and ipsilateral and contralateral mediastinal or supraclavicular nodes	Chemoradiation (cisplatin and etoposide or vinorelbine) Surgery may be offered for IIIa but not IIIb Targeted therapy (alectinib, osimertinib) and/or immunotherapy (pembrolizumab, nivolumab)
IV	Single or multiple extrathoracic metastases	Targeted therapy Immunotherapy Palliative care

## Data Availability

No data were created.
